# A genetically stable rooting protocol for propagating a threatened medicinal plant—*Celastrus paniculatus*

**DOI:** 10.1093/aobpla/pls054

**Published:** 2012-12-31

**Authors:** Mahendra Phulwaria, Manoj K. Rai, Ashok Kumar Patel, Vinod Kataria, N. S. Shekhawat

**Affiliations:** Biotechnology Unit, Department of Botany, Jai Narain Vyas University, Jodhpur 342001, Rajasthan, India

**Keywords:** *Celastrus paniculatus*, *ex vitro* rooting, genetic fidelity, molecular marker, shoot multiplication.

## Abstract

*Celastrus paniculatus*, belonging to the family Celastraceae, is an important medicinal plant of India. Owing to the ever-increasing demand from the pharmaceutical industry, the species is being overexploited, thereby threatening its stock in the wild. Poor seed viability coupled with low germination restricts its propagation through sexual means. Thus, alternative approaches such as *in vitro* techniques are highly desirable for large-scale propagation of this medicinally important plant. Nodal segments, obtained from a 12-year-old mature plant, were used as explants for multiple shoot induction. Shoot multiplication was achieved by repeated transfer of mother explants and subculturing of *in vitro* produced shoot clumps on Murashige and Skoog's (MS) medium supplemented with various concentrations of 6-benzylaminopurine (BAP) alone or in combination with auxin (indole-3-acetic acid (IAA) or α-naphthalene acetic acid (NAA)). The maximum number of shoots (47.75 ± 2.58) was observed on MS medium supplemented with BAP (0.5 mg L^−1^) and IAA (0.1 mg L^−1^). *In vitro* raised shoots were rooted under *ex vitro* conditions after treating them with indole-3-butyric acid (300 mg L^−1^) for 3 min. Over 95 % of plantlets acclimatized successfully. The genetic fidelity of the regenerated plants was assessed using random amplified polymorphic DNA. No polymorphism was detected in regenerated plants and the mother plant, revealing the genetic fidelity of the *in vitro* raised plantlets. The protocol discussed could be effectively employed for large-scale multiplication of *C. paniculatus*. Its commercial application could be realized for the large-scale multiplication and supply to the State Forest Department.

## Abstract (precis)

Nodal segments, obtained from 12 years-old mature plant, were used as explants for *in vitro* propagation of *Celastrus paniculatus*, an important medicinal plant of India. Shoot multiplication was achieved by repeated transfer of mother explants and subculturing of *in vitro* produced shoot clumps on MS medium supplemented with various concentrations of BAP alone or in combination with auxin (IAA or NAA). *In vitro* raised shoots were rooted under *ex vitro* condition. Genetic fidelity of the regenerated plants was assessed using random amplified polymorphic DNA (RAPD).

## Introduction

*Celastrus paniculatus* (known as *Jyotishmati* in Sanskrit and *Malkangni* in Hindi), belonging to the family Celastraceae, is an important medicinal plant of India. Oil obtained from the seeds of this plant is a source of herbal medicine, which is used in the treatment of gout, leprosy, skin diseases, fever, rheumatism, beriberi, sores and neurological disorders (Warrier *et al.* 1994; Lal and Singh 2010). Celapagin, celapanigin, celapanin, celastrine and paniculatine are the alkaloids found in the seed oil which are responsible for making the plant medicinally highly potent (Parimala *et al.* 2009). Several studies have confirmed the memory- and grasping-power-boosting properties of *C. paniculatus* (Nair and Seeni 2001; Godkar *et al.* 2004). Apart from these therapeutic applications, its roots are also used in the treatment of cancerous tumours (Parotta 2001).

Conventionally, *C. paniculatus* is propagated mainly through the seeds. However, the viability and germination (11.5 %) of the seeds are poor (Rekha *et al.* 2005). Increasing human and livestock populations have affected the status of wild plants, particularly those used in medicines (Singh *et al.* 2009). Owing to its pharmaceutical importance, this species has been overexploited and is now considered a threatened species (Martin *et al.* 2006; Lal and Singh 2010; Raju and Prasad 2010). Thus, there is an urgent need to conserve the natural stock and multiply the plants on a larger scale to reduce the dependence on the forests for the supply of raw drugs. In the past three to four decades, plant tissue culture has been exploited as a valuable tool for the conservation and large-scale propagation of medicinally important and endangered plants (Phulwaria *et al.* 2012*a*, 2013; Thiyagarajan and Venkatachalam 2012). However, the influence of culture conditions, for instance, culture media, type of explant, successive transfer of culture, temperature, pH, etc., leads to genomic changes in the *in vitro* raised plantlets. Thus, analysis of the genetic homogeneity of tissue culture raised plants is a prerequisite for their exploitation on a commercial scale (Rai, M. K. *et al.* 2012). DNA-based molecular markers are powerful tools for analysis of the genetic fidelity of micropropagated plantlets (Ahmad and Anis 2011; Sharma *et al.* 2011).

The present paper concerns an improved and reproducible protocol for large-scale propagation of *C. paniculatus.* The multiplication rate achieved in the present investigation is better than that in earlier reports on the same taxon (Nair and Seeni 2001; Arya *et al.* 2002; Sharada *et al.* 2003; Martin *et al.* 2006; Rao and Purohit 2006; Raju and Prasad 2007; Lal and Singh 2010). To save time and reduce labour cost, efforts were also made to optimize the most favourable conditions for *ex vitro* rooting. Furthermore, to confirm the genetic fidelity, regenerated plants underwent molecular analysis using DNA-based molecular markers.

## Materials and methods

### Explant preparation and surface sterilization

An approximately 12-year-old plant of *C. paniculatus*, maintained in the field of the Biotechnology Unit, JNV University, Jodhpur, Rajasthan, India, was used as the mother plant to collect the explants. Juvenile shoots obtained from freshly emerged sprouts were collected and nodal segments (3–5 cm) were excised. Prior to surface sterilization using 0.1 % (w/v) HgCl_2_ (Hi-Media, India) for 5 min under aseptic conditions, explants were treated with 0.1 % (w/v) Bavistin (BASF India Limited, Mumbai, Maharashtra, India) for 5–7 min to reduce the chance of fungal contamination. Explants (2–3 cm) were finally rinsed (5–6 times) with sterile double-distilled water to remove any traces of the disinfectant.

### Nutrient media and culture conditions

Murashige and Skoog's (MS; Murashige and Skoog 1962) medium supplemented with sucrose (3 %) and additives (50 mg L^−1^ ascorbic acid, 25 mg L^−1^ adenine sulfate, 25 mg L^−1^ arginine and 25 mg L^−1^ citric acid), as described by Arya *et al.* (2002), was used for culture initiation. Agar-agar (Hi-Media, India) was added to the culture media as a gelling agent at a concentration of 0.8 % (w/v). The pH of the medium was adjusted to 5.8 ± 0.02 and the medium was then autoclaved for 15 min at 121 °C. Cultures were maintained at 28 ± 2 °C under a 14 h day^−1^ photoperiod with a light intensity of 40–50 µmol m^−2^s^−1^ photon flux density, provided using cool white fluorescent lamps (Philips, India).

### Shoot multiplication

To study the effect of plant growth regulators (PGRs) on bud break, nodal explants were cultured on MS medium containing different kinds (6-benzylaminopurine (BAP) or kinetin (Kin)) and concentrations (0.0, 1.0, 2.0, 3.0 or 5.0 mg L^−1^) of cytokinins. Cultures showing bud break were further multiplied using two approaches: (i) *in vitro* produced axillary shoots were excised and the mother explants were transferred repetitively for four passages (at 3-week intervals) onto MS medium containing 1.0 mg L^−1^ BAP plus additives; and (ii) shoot clumps (each containing 4–5 shoots) were excised from mother cultures and transferred onto MS medium containing BAP alone (0.0, 0.25, 0.5, 1.0 or 1.5 mg L^−1^) or a combination of an optimized concentration of BAP (0.5 mg L^−1^) with different concentrations (0.0, 0.05, 0.1, 0.2 or 0.3 mg L^−1^) of indole-3-acetic acid (IAA) or α-naphthalene acetic acid (NAA) for shoot multiplication and elongation.

### *Ex vitro* rooting of micropropagated shoots

To induce roots under *ex vitro* conditions, shoots (5–8 cm long) were excised from *in vitro* developed multiple shoots and the basal ends were treated with different concentrations (0.0, 100, 200, 300, 400 or 500 mg L^−1^) of indole-3-butyric acid (IBA) or NAA for 3 min. The treated shoots were transferred to bottles containing sterile soil-rite (a mixture of horticulture-grade perlite with Irish peat-moss and exfoliated vermiculite; supplied by Kel Perlite, Bangalore, Karnataka, India) moistened with an aqueous solution of one-quarter strength of MS basal salts. These bottles were kept in the greenhouse near the pad section in order to maintain high relative humidity (80–90 %) and low-temperature conditions (28 ± 2 °C).

### Hardening of *in vitro* produced plantlets

After root initiation, the caps of bottles were gradually opened over a period of 2 weeks and finally removed. Bottles containing plantlets were then shifted from the pad section towards the fan section to provide growing conditions of low humidity (60–70 %) and high temperature (32 ± 2 °C). After 4–5 weeks, acclimatized plantlets were transferred to poly-bags containing a mixture of organic manure, clay and sand (1 : 1 : 1). Such plantlets were kept in the greenhouse for 7–8 weeks, shifted to the nursery and finally transferred to the field for the evaluation of percentage survival of plants.

### Assessment of genetic fidelity using random amplified polymorphic DNA analysis

The genetic stability of the 13 randomly selected *in vitro* regenerated plants was assessed using polymerase chain reaction (PCR)-based random amplified polymorphic DNA (RAPD) analysis. Genomic DNA was extracted from the fresh juvenile leaves of the mother plant and *in vitro* regenerated plants by following the cetyl trimethyl ammonium bromide (CTAB) method (Doyle and Doyle 1990). Extracted DNA was quantified using a UV–Visible Elico spectrophotometer and the quality of DNA was tested following electrophoresis on a 0.8 % agarose gel. After preliminary screening of 25 primers, 16 RAPD primers were selected for genetic fidelity of tissue culture raised plants (Table 4). The PCR was performed in a 15 µL reaction mixture containing 2.0 µL of template DNA (50–60 ng), 1.5 µL of 10× PCR buffer (Bangalore Genei, Bangalore, Karnataka, India), 1.5 µL of MgCl_2_ (2.5 mM; Bangalore Genei), 0.3 µL of deoxyribonucleotide triphosphates (10 mM; Bangalore Genei), 0.8 µL of random primer (10 µM; Integrated DNA Technologies Inc., India), 0.5 µL of three unit Taq polymerase (Bangalore Genei) and 8.6 µL of sterile distilled water. DNA amplification was carried out in a thermal cycler (Eppendorf 5331, Germany). The PCR programme consisted of an initial denaturation for 4 min at 94 °C, then 40 cycles of 30 s denaturation at 94 °C, 1.20 min annealing at *T*_a_ °C and 1.30 min extension at 72 °C with a final extension at 72 °C for 10 min. The annealing temperature (*T*_a_) was kept at 2 °C below the melting temperature (*T*_m_) of that particular primer sequence. Amplification with each primer was repeated twice to confirm the reproducibility of the results. The amplification products for all samples were resolved on a 1.4 % agarose (A9539; Sigma, St Louis, MO, USA) gel using 1× Tris/borate/EDTA buffer and stained with ethidium bromide (0.25 µg mL^−1^). Gels were visualized using a gel documentation system (Syngene Gel Doc; Syngene, Synoptics Ltd, UK). The size of the amplicons was estimated by comparison with a 100-bp DNA ladder (Bangalore Genei).

### Experimental design and statistical analysis

All the experiments were conducted with a minimum of 20 replicates per treatment. Each explant was taken as a replication and all the experiments were repeated three times. The results were expressed as the mean ± SD for all experiments. The data were subjected to statistical analysis using one-way analysis of variance, and the significance between means was assessed by Duncan's multiple range test (DMRT) at *P* < 0.05.

## Results and discussion

### Explant selection and shoot induction

**Fig. 1:**
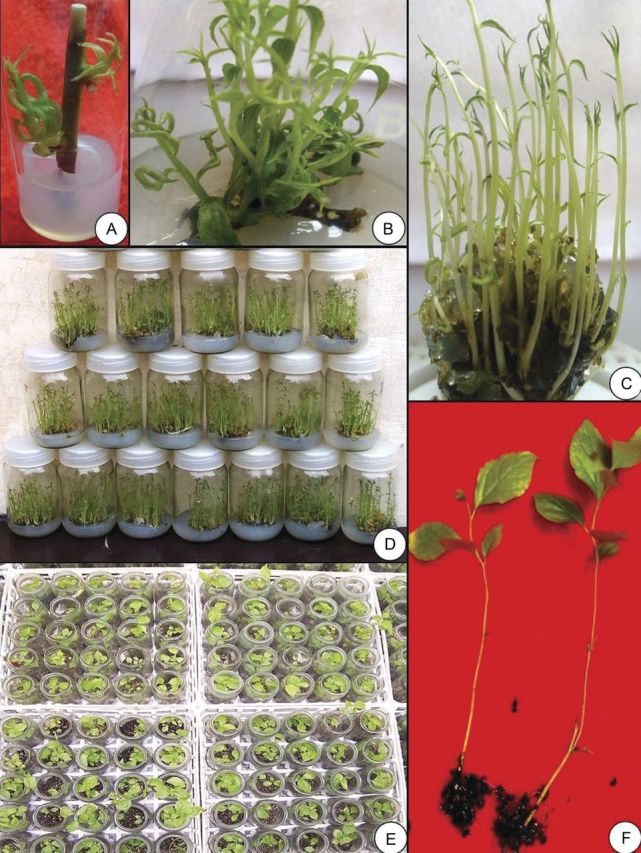
**Micropropagation of *C. paniculatus*.** (A) Nodal explants showing bud break on MS medium containing 2.0 mg L^−1^ BAP after 1–2 weeks of culture. (B) Multiplication of shoots after repeated transfer of mother explant after two passages on MS + 1.0 mg L^−1^ BAP. (C) Multiplication of shoots from the transfer of excised shoot clumps (4–5 shoots) to MS + 0.5 mg L^−1^ BAP + 0.1 mg L^−1^ IAA after 4 weeks of transfer. (D) Large-scale shoot proliferation on MS + 0.5 mg L^−1^ BAP + 0.1 mg L^−1^ IAA after 4 weeks of transfer. (E) *Ex vitro* rooted shoots after treatment with 300 mg L^−1^ IBA for 3 min after 3–4 weeks. (F) Micropropagated plants in the greenhouse near the pad section.

In the present study, we established axenic cultures of *C. paniculatus* from nodal explants derived from an approximately 12-year-old plant. Culture responsiveness was found to be better for the explants collected during March–April. However, those collected during other periods were found to be less responsive and exhibited more contamination (data not presented). The seasonal effect on culture establishment has also been reported in *Azadirachta indica* (Arora *et al.* 2010) and *Salvadora persica* (Phulwaria *et al.* 2011). Bud break was observed for the explants cultured on MS medium containing cytokinins (BAP or Kin) after 1–2 weeks. Among the cytokinins and their concentrations tested, BAP (2 mg L^−1^) was found to be most favourable for shoot bud induction (Fig. 1A; Table 1). Kinetin, however, was found to be less effective in causing bud break and in the induction of multiple shoots. The effectiveness of BAP in shoot proliferation has been well documented for a number of plant species (Kumar *et al.* 2010; Singh and Tiwari 2012).

**Table 1:** Effect of cytokinins (BAP or Kin) on multiple shoot induction from nodal explants of *C. paniculatus*.

BAP (mg L^−1^)	Kin (mg L^−1^)	Percentage of bud breaking	Shoot number (mean ± SD)	Shoot length (cm) (mean ± SD)
0.0	0.0	0.0	0.0	0.0
1.0	–	50.5^e^	1.25 ± 0.43^f^	1.17 ± 0.20^e^
2.0	–	80.0^a^	3.25 ± 0.82^a^	2.77 ± 0.17^a^
3.0	–	70.0^b^	2.50 ± 0.50^b^	2.25 ± 0.22^b^
5.0	–	65.5^c^	1.60 ± 0.32^cd^	1.42 ± 0.27^c^
–	1.0	25.5^h^	1.20 ± 0.40^g^	1.06 ± 0.30^g^
–	2.0	55.0^d^	1.75 ± 0.43^c^	1.24 ± 0.11^d^
–	3.0	40.5^f^	1.25 ± 0.23^fg^	1.15 ± 0.28^ef^
–	5.0	35.0^g^	1.40 ± 0.48^e^	1.01 ± 0.35^h^

Means in each column followed by the same superscript letters are not significantly different according to DMRT at *P* < 0.05.

### Shoot multiplication

**Fig. 2:**
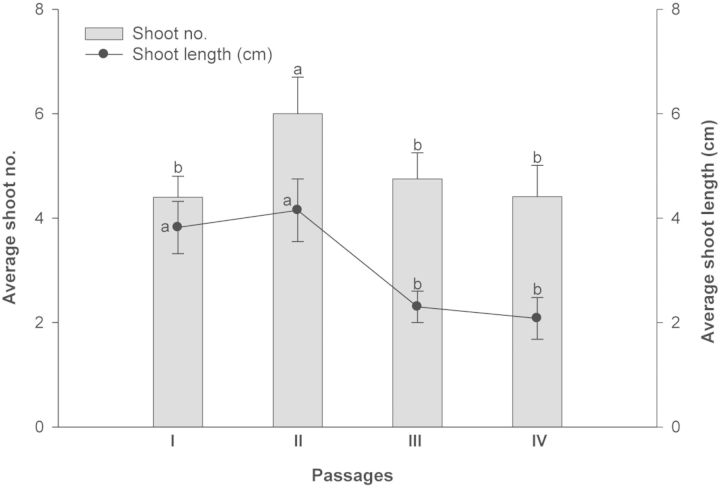
**Shoot multiplication by repeated transfer of mother explant for different passages on MS + 1.0 mg L^−1^ BAP + additives.** Mean values sharing the same letter do not differ significantly (*P* < 0.05) according to DMRT.

After excising multiple shoots, when mother explants were transferred to the fresh shoot multiplication medium (MS medium + 1.0 mg L^−1^ BAP), they proliferated significantly during the next two subcultures and reduced thereafter (Fig. 2). The highest number of shoots (6–7 shoots per explant) was produced during these two passages (Fig. 1B). Repeated transfer of the original explants was suggested as an efficient technique for rejuvenation and reinvigoration of *in vitro* cultures (Sanchez *et al.* 1997), which was further supported by subsequent reports on different plants (Abraham *et al.* 2010; Ram and Shekhawat 2011; Phulwaria *et al*. 2012*b*).

To improve shoot multiplication in the subsequent culture, shoot clumps (4–5 shoots) were excised and subcultured on MS medium containing different concentrations of BAP, either alone or in combination with IAA. Of the combinations studied, the medium supplemented with 0.5 mg L^−1^ BAP + 0.1 mg L^−1^ IAA was found to be most suitable for shoot multiplication and growth (Table 2; Fig. 1C). This multiplication rate (47.75 ± 2.58) appears to be superior compared with previous reports published for the same plant species (Nair and Seeni 2001; Arya *et al.* 2002; Sharada *et al.* 2003; Martin *et al.* 2006; Rao and Purohit 2006; Raju and Prasad 2007; Lal and Singh 2010). Regular subculture on a similar kind of medium at 3- to 4-week intervals, coupled with this approach, increased the multiplication rate by 8- to 10-fold (Fig. 1D). This finding is in accordance with earlier reports for different plants (Ram and Shekhawat 2011; Phulwaria *et al.* 2012*a*, *b*).

**Table 2:** Effect of PGRs on shoot multiplication of *C. paniculatus*.

BAP (mg L^−1^)	IAA (mg L^−1^)	NAA (mg L^−1^)	Number of shoots (mean ± SD)	Shoot length (cm) (mean ± SD)
0.0	0.0	0.0	0.0	0.0
0.25	–	–	19.10 ± 1.06^k^	2.32 ± 0.34^ijk^
0.5	–	–	29.75 ± 1.40^e^	4.10 ± 0.70^ef^
1.0	–	–	24.54 ± 1.22^fg^	3.12 ± 0.64^g^
1.5	–	–	20.40 ± 2.90^hi^	2.20 ± 0.60^ij^
0.5	0.05	–	32.32 ± 2.50^c^	5.46 ± 0.28^cd^
0.5	0.1	–	47.75 ± 2.58^a^	8.50 ± 1.10^a^
0.5	0.2	–	35.50 ± 1.41^b^	6.70 ± 0.20^b^
0.5	0.3	–	30.12 ± 2.60^d^	5.96 ± 0.38^c^
0.5	–	0.05	22.64 ± 1.36^h^	3.21 ± 0.35^gh^
0.5	–	0.1	28.50 ± 3.41^de^	5.70 ± 1.20^b^
0.5	–	0.2	23.52 ± 2.10^fg^	3.96 ± 0.98^e^
0.5	–	0.3	20.64 ± 1.82^ij^	2.64 ± 0.34^i^

Means in each column followed by the same superscript letters are not significantly different according to DMRT at *P* < 0.05.

### *Ex vitro* rooting and acclimatization of plantlets

In general, plants developed through *ex vitro* rooting have lateral roots similar to the natural root system, resulting in better hardening and improved survival than in those developed from *in vitro* rooting (Dhavala and Rathore 2010; Yan *et al.* 2010; Phulwaria *et al.* 2012*a*). Compared with NAA, IBA was found to be a better auxin for root induction. One hundred per cent rooting was observed when shoots were treated with 300 mg L^−1^ IBA for 3 min (Fig. 1E). Although lower concentration of IBA could induce roots in all microcuttings, the number and length of roots declined (Table 3). One of the major advantages of *ex vitro* rooting is that plantlets do not need any additional acclimatization prior to transplanting in the field conditions (Yan *et al*. 2010).

**Table 3:** Effect of auxins (IBA or NAA) on *ex vitro* rooting of micropropagated shoots of *C. paniculatus*.

IBA (mg L^−1^)	NAA (mg L^−1^)	Percentage of rooting	Number of roots (mean ± SD)	Root length (cm) (mean ± SD)
0.0	0.0	0.0	0.0	0.0
100	–	90.2^b^	5.21 ± 0.34^c^	1.85 ± 0.64^de^
200	–	100^a^	6.25 ± 0.82^b^	2.55 ± 0.36^bc^
300	–	100^a^	8.42 ± 0.70^a^	3.25 ± 0.82^a^
400	–	81.3^c^	4.25 ± 0.82^cd^	1.87 ± 0.21^d^
500	–	70.5^e^	3.52 ± 0.56^de^	1.25 ± 0.62^g^
–	100	50.2^h^	1.90 ± 0.83^gh^	1.10 ± 0.20^h^
–	200	80.6^cd^	3.40 ± 0.41^de^	2.90 ± 0.30^b^
–	300	78.2^de^	2.95 ± 0.84^f^	2.25 ± 0.82^c^
–	400	62.4^f^	1.92 ± 0.21^g^	1.82 ± 0.32^def^
–	500	57.8^g^	1.10 ± 0.43^i^	1.23 ± 0.46^gh^

Treatment duration: 3 min. Means in each column followed by the same superscript letters are not significantly different according to DMRT at *P* < 0.05.

**Fig. 3:**
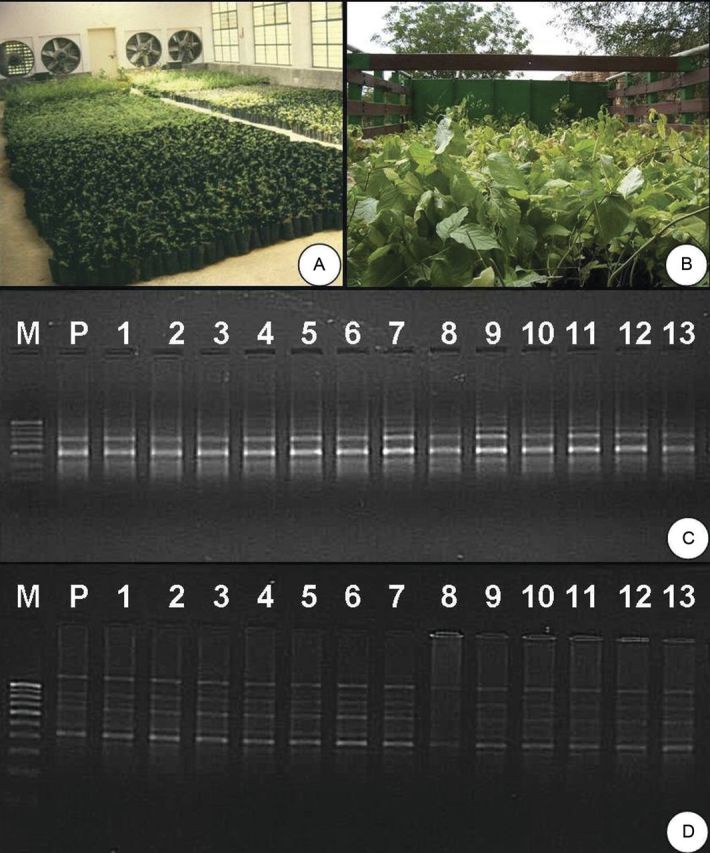
**Acclimatization of plants and DNA amplification obtained with primers.** (A) Micropropagated plants transferred to poly-bags in the greenhouse near the fan section. (B) Fully hardened plantlets loaded for transportation to the State Forest Department. (C and D) DNA amplification obtained with primers (C) OPG-07 and (D) OPQ-07. Lane P, DNA from the mother plant; lanes 1–13, DNA from micropropagated plants.

Rooted plantlets were transferred to the greenhouse for secondary hardening (Fig. 1F) and such hardened plantlets were finally transferred to the poly-bags containing sand, garden soil and organic manure (1 : 1 : 1) (Fig. 3A). These plantlets were kept in the greenhouse for 50–60 days, shifted to the nursery and finally transferred to the field for evaluation. Using the described protocol, a large number of plants have been produced and transported to the State Forest Department for field transfer (Fig. 3B).

### Assessment of the genetic fidelity of micropropagated plants

Only 16 of the 25 RAPD 10-mer primers screened were found to be polymorphic, producing 65 amplicons in total, ranging from 300 to 1200 bp in size. The number of bands in the selected primers varied from three (OPA-13, OPQ-17) to six (OPQ-07), with a mean of 4.1 bands per RAPD primer (Table 4). The RAPD amplification patterns obtained with primers OPG-07 (Fig. 3C) and OPQ-07 (Fig. 3D) clearly suggest that the micropropagated plants were genetically identical to the mother plant and no variation was induced during *in vitro* propagation. Molecular markers, mainly RAPD, have been widely used for testing the genetic fidelity among *in vitro* regenerated plantlets in a number of plant species (Ahmad and Anis 2011; Ramesh *et al.* 2011; Sharma *et al.* 2011; Rai, G. K. *et al.* 2012; Savita *et al.* 2012; Singh *et al.* 2012), owing to their simplicity, cost-effectiveness and lower DNA requirement for analysis (Asthana *et al.* 2011).

**Table 4:** RAPD primers used for testing the genetic fidelity of micropropagated plants of *C. paniculatus*.

Primer code	Primer sequence 5′–3′ (length)	Number of scorable bands	Range of amplification (bp)
OPC-02	GTGAGGCGTC	5	400–1200
OPC-05	GATGACCGCC	4	300–900
OPA-13	CAGCACCCAC	3	400–1100
OPG-03	GAGCCCTCCA	4	300–1000
OPH-05	AGTCGTCCCC	5	300–900
OPG-07	GAACCTGCGG	4	300–1000
OPAB-18	CTGGCGTGTC	4	400–1200
OPB-07	GGTGACGCAG	4	300–1000
OPQ-07	CCCCGATGGT	6	400–1200
OPA-09	GGGTAACGCC	5	300–1000
OPH-15	AATGGCGCAG	4	400–1100
OPG-15	ACTGGGACTC	4	300–1000
OPAB-12	CCTGTACCGA	4	400–1200
OPQ-17	GAAGCCCTTG	3	300–900
OPG-09	CTGACGTCAC	3	300–1000
OPC-04	CCGCATCTAC	3	400–1000

## Conclusion and forward look

The improved micropropagation protocol discussed here could be exploited for large-scale multiplication of *C. paniculatus*, a threatened and medicinally important plant species. Continuous supply of plants through micropropagation would reduce the pressure on the species' natural population and thus help conserve it. The technique of inducing roots under *ex vitro* conditions has made the discussed protocol more efficient and economical owing to the improved survival. Apart from being rapid, the protocol was found to cause no genetic variation among the regenerants and hence could be of great practical utility.

## Sources of funding

The work was funded by the University Grants Commission (UGC), New Delhi, through a Post Doctoral Fellowship and Dr D.S. Kothari Post Doctoral Fellowship to M.P. and M.K.R. Financial assistance provided by the Council of Scientific and Industrial Research (CSIR), New Delhi, to A.K.P. is gratefully acknowledged.

## Contributions by the authors

M.P. designed and performed the experiments and wrote the first draft of the manuscript. M.K.R. analysed data and organized it in figures and tables. A.K.P. performed some tissue culture experiments. V.K. and N.S.S. edited the final version of the manuscript.

## Conflict of interest statement

None declared.
